# Life experiences vs digital inclusion: Factors influencing sense of security and their evolutions in China

**DOI:** 10.1371/journal.pone.0330667

**Published:** 2025-08-28

**Authors:** Lijuan Zhao

**Affiliations:** School of Public Administration, Central South University, Changsha, China; Cardiff Metropolitan University - Llandaff Campus: Cardiff Metropolitan University, UNITED KINGDOM OF GREAT BRITAIN AND NORTHERN IRELAND

## Abstract

**Background:**

Digital technology is reshaping and enrich the types of social risks, making the lack of sense of security a widespread phenomenon. The predictors and enhancement strategies for the sense of security have drawn academic attention. However, there is still a debate on the relative effects of objective life experience and media construction on sense of security and how these influences evolve over time. Therefore, this study empirically examines the impact of digital media use and real-life experiences and their evolving mechanisms.

**Methods:**

This study based on data from CSS in 2013, 2017, and 2021, explored the impact and evolution of life experiences and digital inclusion on sense of security from the perspectives of experiential shaping and media construction.

**Results:**

Results indicate that negative life experiences have a significant negative impact on sense of security, which first decreases but then increases over time. Digital inclusion also has a significant negative impact on sense of security, and this trend diminishes over time. Moreover, the results of relative importance show that despite digital inclusion’s growing explanatory power, life experiences remain dominant, which means the current level of digital inclusion still cannot shake the decisive role of personal experiences. The educational differences in the impact of digital inclusion on sense of security suggest that education and digital literacy can mitigate the negative effects of digital inclusion, potentially turning them into positive influences.

**Conclusion:**

The findings indicate that in the process of digital society risk governance, it is also necessary to closely monitor the real-life domains of the public in order to achieve risk governance in both online and offline spaces.

## 1. Introduction

Sense of security, as a subjective psychological experience, is not equivalent to being safe. On the one hand, the harsh reality of various social risks and significant safety incidents, along with personal experiences, impinge upon people’s perception and experience of their surround safety. On the other hand, the development of internet technology and multimedia platforms, coupled with the spread of false reports and exaggerated expressions in online public discourse, further intensifies the anxiety and concerns about the current safety. The real challenges of safety issues and the constructed narratives in the media are the two major threats affecting sense of security. Digital technology has been the most pivotal element since the 21st century, transforming not only the spaces in which people produce and live but also their social interactions, and exerting a profound influence on their cognition and social psychology.

The transition towards informatization and digitization in China has had a significant impact on the societal environment and types of risks, thereby shaping individuals’ social perception, including sense of security. Compared with traditional mass media era, the characteristics of risks in digital societies are primarily reflected in the following aspects: changed the generation and dissemination of risks, normless behaviors in anonymous spaces, and exacerbating social inequality of the digital divide. Digitalization has made various risks and discordant elements more easily triggered. The absence of sense of security has become a widespread social phenomenon, prompting scholars to investigate its predictors and enhancement strategies. A contentious issue in this dominance is the relative effects and temporal trends of objective experiences compared with media constructions. Given that the previous studies have typically focused either on direct experiences or on media construction, the conclusions drawn from such single-perspective research lack explanatory power for the current security issues in the context of the integration of digital and physical experiences. Hence, this research aims to explore the impact of digital media usage and real-life experiences on sense of security and their evolutions through empirical analysis.

## 2. Research background and hypotheses

### 2.1 Experience shaping perspective: negative life experiences and sense of security

The experience shaping theory of sense of security posits that an individual’s social experiences and life experiences significantly influence its formation of sense of security [1–2]. Cognition and judgment of safety conditions reflect objective safety issues or potential risk threats, which are closely linked with past experiences. In other words, the way people perceive and evaluate the safety of their surroundings or future prospects is deeply rooted in their accumulated experiences. Weng (2010) pointed out that people often extend their limited, “constructed” life experiences to understand macro society, forming social cognition and attitudes [3]. As a result, their past encounters, whether positive or negative, shape their overall understanding and perception of the world around them. An individual’s judgment of the safety of their current environment or future situation is often based on “memories of past dangerous experiences or expectations of future risk realizations.” [4] Personal experiences, stored as information in memory, are reactivated during judgement-making, influencing social cognition and evaluation. When faced with new situations or decisions, the brain draws upon these stored memories to make sense of the present and predict the future. The crucial role of personal experiences in forming the sense of security is unquestionable [5–7]. Negative life experiences were the key part of the latent construct of “uncertainty and unpredictability” from the life history theory perspective [8,9]. Groups that have suffered physical or psychological harm, lived in adverse conditions, or experienced major crisis events often exhibit a stronger sense of insecurity [10–13].

Social and economic transformation in China has amplified the complexity and uncertainty of risks, challenging traditional cognitive approaches. Political and economic reforms, coupled with socio-economic growth, have led to a rise in social risks, characterized by pronounced class stratification and intensifying social contradictions. These changes have created a more intricates and volatile social landscape. Habermas described modern risks as arising from human knowledge and technological advancements, which have a far-reaching impact on the world [14]. These risks exhibit a dual nature of objectivity and subjectivity, reality and virtuality, and macroscopic and openness. As Beck et al. (2010) noted, heightened risk awareness itself can become a risk, as people construct elusive risks out of future concern [15]. In the past, practical risks were limited, and life experiences played a significant role in risk assessment and security perceptions. However, with the rapid changes and the emergence of new types of risks, the role of life experiences has been somewhat diminished. Under the Party Central Committee’s guidance [16], a social risk governance framework characterized by co-building, co-governing, and co-sharing risk regulation capabilities has been established. As a result, risk regulation capabilities have been significantly improved, effectively diminishing the influence of real-life risks on the sense of security. On this basis, we propose the following hypotheses:

**H1-1**. *Negative life experiences have a negative impact on the public sense of security;*

**H1-2**. *Over time, the adverse impact of life experiences on the public sense of security shows a weakening trend*.

### 2.2 Media construction perspective: digital inclusion and sense of security

The media construction theory of sense of security refers to the constructability of risks and the cultivation effect of media [17,18]. Beck emphasized that risks are anticipatory, not actual disasters [19]. Historically, risk perceptions stemmed from personal experiences but now heavily rely on media, especially with the rise of mass communication and the digital age [20]. The evolution of risk construction media has progressed from traditional to digital forms, impacting the sense of security. The digital transformation introduces both subjective and objective risk domains, the latter of which can cause widespread insecurity in digital technology use [21]. Research should pivot from the ‘quasi-environment’ to ‘digital inclusion,’ emphasizing public participatory agency [22] and suggest that social risks in the digital era follow a path from objective existence to subjective cognition and behavioral choice, advocating for a participatory approach to risk perception [14,20].

Scholars have observed public engagement and behavioral responses to Internet use. Gong and Fan (2021) discovered that increased exposure to new media is correlated with lower risk acceptance and heightened risk perception [23]. Internet use, as an objective behavior, amplifies exposure to risk information, thereby increasing perceived security risks through social interaction and information acquisition [24–26]. Despite their focus on non-informational aspects of the internet, their conclusions still consider information dissemination and negativity bias. Data from the Chinese General Social Survey 2017 indicate that approximately 120 minutes daily are spent daily on news via the internet and apps such as Weibo and WeChat, underscoring the significant role of the informational function of digital media. This suggests that digital inclusion negatively influences the sense of security.

Owing to digital imbalance, relatively low digital literacy, and lack of digital norms in China, public subjectivity has activated consciousness with hindered growth, blending irrationality and rationality. Early media theories stressed irrational audience judgment, whereas digital inclusion emphasized rational public assessment. As society rationalizes politically, legally, culturally, and economically, individual thinking patterns evolve. With balanced digital development, improved online norms, and heightened digital literacy, the negative effects of digitally activated subjectivity diminish. Hypotheses follow.

**H2-1**. *Digital inclusion has a negative impact on the public sense of security;*

**H2-2**. *Over time, the negative impact of digital inclusion on the public sense of security shows a weakening trend*.

### 2.3 Life experiences vs digital inclusion: whose impact is stronger?

The sense of security is shaped by real predicaments and potential threats. As the Digital China Strategy progresses and the digital economy has expanded, the basis for sense of security has evolved. In the digital realm, sense of security extends beyond personal experiences and direct interactions, necessitating a digital mindset that reevaluates the past and present to inform future expectations. Traditional societies and the early internet eras confined risks by geography and time, but the digital era transcends these limits, moving risks into the virtual sphere. Personal and social experiences are no longer the sole benchmarks for security assessments, as online media has become a primary source of risk information [4,27]. Digital media influences not only information dissemination but also social cognition and attitudes through digital behaviors [28,29]. Weng and Zhang (2018) highlighted the Internet’s impact on public conflict awareness, with online information outweighing personal frustration experiences [30]. With the rise of digital societies and smart cities, digital inclusion is essential for survival, underscoring its significance in the new paradigm. Therefore, with the establishment of a panoramic digital society and smart cities, digital inclusion has become a basic requirement for survival, and this transformation of survival and lifestyle further highlights the importance of digital inclusion. Thus, the third hypothesis is proposed:

**H3**. *With the full coverage of digital life scenarios, compared with life experiences, digital inclusion has a greater impact on the public sense of security.*

Previous studies have made progress in understanding the sense of security, yet gaps remain. First, there is a scarcity of comprehensive national studies on this topic, leading to fragmented findings. Second, discussions on digital security are often limited to personal or media-centric views, overlooking broader temporal analyses. Third, current theories do not fully capture the multifaceted nature of security in the digital era. This research leverages the CSS database to analyze the dynamic interplay between life experiences, digital engagement, and security perceptions, assessing their comparative influence.

## 3. Data and methods

### 3.1 Data source

This study adopts the samples from the Chinese Social Survey (CSS) conducted by the Institute of Sociology, Chinese Academy of Social Sciences, which started in 2005. The CSS uses a multi-order stratified PPS random sample to best represent all Chinese, and all participants were requested to provide written informed consent before participating in the survey. Given our goals, the database has one advantage: the CSS has included modules related to “life experiences,” “digital inclusion,” and “sense of security”, which are highly coherent and provide strong data support for testing the impact of life experiences and digital inclusion on sense of security, as well as the evolving mechanisms of their influence over time.

This study constructs a sample for empirical analysis using the latest three waves in 2013, 2017, and 2021, constructing a mixed cross-sectional database. After eliminating key variables containing missing values, a total of 27,464 samples were finally included, with the sample sizes for 2013, 2017, and 2021 of 9,257, 9,470, and 8,737, respectively. The dataset could be found in Supporting Information S1 Data.

### 3.2 Measurements

*Sense of security (SS)* was measured with two indicators. The first is the overall sense of security, which was the main dependent variable and examined by the average score of the respondents’ self-assessment of seven types, including personal, financial, medical, traffic, food, labor, and information security. The question in CSS is “Respondents’ evaluation of the above 7 kins of security”, and the options are “1= very unsafe, 2=not very safe, 3=relatively safe, 4=very safe”. We use the average score as the indicator of sense of security, with a range of 1–4. The second is an alternative indicator for the overall sense of security (*OSS*), which is used for robustness tests. According to previous research [31] and the CSS questionnaire, it is measured by a single-item question “Respondents’ evaluation of the current overall social security situation: very unsafe, not very safe, relatively safe, and very safe,” with a value ranging from 1–4. The higher the value is, the greater the sense of security.

*Life Experiences (LE)* refers to the events or problems individuals encounter in their daily lives and work, from which they can gauge the surrounding social environment and make assessments and judgments about the present or future. In accordance with Yang and Lian (2015), this study operationalizes life experiences as the number of problems an individual has encountered over a certain period [32]. For example, in the CSS 2021 survey, the item is “In the past year, have you encountered any of the following life problems? Poor housing conditions, high costs of children’s education, heavy burdens of raising children, disharmonious family relationships, high medical expenses, rising prices affecting life, low family income, family members being unemployed, jobless, and serious residential environmental issues,” with options “1=Yes” and “0=No.” This study measures the cumulative degree of negative life experiences by adding up the above options; the higher the value is, the more negative life experiences. Similarly, the CSS 2013 and 2017 waves are also processed. To ensure the robustness of the results, this study also adopts “major life events (*MLE*)” as an alternative indicator. In CSS, the item regarding “major life events” is “In the past year, have you encountered any of the following events? Seeking medical treatment, children attending school, job seeking and employment, asking for a raise or promotion, litigation, doing business.” There are also “1=Yes” and “0=No”, and the same calculation method as the above. The higher the value, the more major events there are.

*Digital Inclusion (DI)* refers to the acceptance of digital technology, the willingness and ability to use digital technology, thereby adapting to a digital lifestyle. Liu (2021) suggested that the operational definition of digital inclusion should be closely centered around “the use of digital technology in daily life.” [22] Based on Maslow’s hierarchy of needs, digital inclusion can be divided into three dimensions: “primary inclusion,” “medium inclusion,” and “advanced inclusion,” highlighting the Internet access and digital skill application behaviors driven by the satisfaction of needs. Therefore, combining this perspective with the relevant items in the CSS, after multiple rounds of statistics and factor analysis, three dimensions and corresponding items are shown in Table 1.

**Table 1:** Description of digital inclusion.

Dimension	Definition	Items	Options	Cronbach’s α	range
primary digital inclusion (*PDI*)	Whether individuals have access to or the ability to use the Internet, which is the basic requirement for digital inclusion	Now that the internet is widely available, do you use mobile phones and computers to access the Internet?	Online = 1, Not online = 0.	—	0-1
medium digital inclusion (*MDI*)	individuals using the Internet or digital media to communicate with others, or for leisure activities such as watching videos	How often do you go online for leisure and entertainment, chatting and making friends, etc.?	0 = Never,..., 5 = Almost every day.	0.97	0-1
advanced digital inclusion (*ADI*)	With the full coverage of digital life scenarios, the ability to complete daily activities such as online learning, online office, online transactions, and financial management through the Internet represents an advanced stage of digital inclusion	How often do you go online for working, learning and education, shopping, investment and financial management, etc.?	0 = Never,..., 5 = Almost every day	0.98	0-1
Digital inclusion	DI=1×PDI+2×MDI+3×ADI			0.85	0-6

*Note*: The table only shows the relevant items from CSS 2021; the items from 2013 and 2017 are similar, with Cronbach’s α coefficients all above 0.8. To achieve comparability across waves, each indicator has undergone standardization processing, with the value range is 0–1.

Due to slight changes of items in three waves, the scores for the three indicators of primary inclusion, medium inclusion, and advanced inclusion for each wave were subjected to min-max normalization processing, converting the indicators to a range between 0 and 1. The purpose of normalization here is to preserve the heterogeneity that individuals may have on various indicators, thereby obtaining a dimensionless comprehensive indicator.

Table 1 shows that the reliability coefficients for each dimension and the entire indicator are above 0.8. Moreover, the means of the *PDI* (0.46), *MDI* (0.34), and *ADI* (0.32) gradually decrease, which is in line with the common sense and life practices. Due to the increasing difficulty of inclusion, when calculating the degree of digital inclusion, different weights are assigned to the three dimensions following existing practices [22]. The equation is: DI=1×PDI+2×MDI+3×ADI.

*Covariates.* Based on previous research [33], the covariates included sex(female = 1), age, hukou (rural = 1), marital status(marriage = 1), residence(urban = 1), educational level (primary and below = 1, high school = 2, bachelor’s degree and above = 3), CPC(CPC = 1), work(worked = 1), area (northeastern = 1, eastern = 2, central = 3, western = 4), and survey year (2013 = 1, 2017 = 2, 2021 = 3). Moreover, considering the potential non-linear influence of age and income, the squared term of age divided by 100 and the squared income are also included in the final analysis. The details of all the variables were presented in Table 2.

**Table 2:** Summary results of the samples.

variables	N	Mean(%)	variables	N	Mean(%)
*SS*	27464	2.92(0.49)	hukou (%)	27464	
*DI*	27464	2.09(2.38)	rural	18452	67.19
*LE (negative)*	27464	0.25(0.20)	non-rural	9012	32.81
Year (%)	27464		residence (%)	27464	
2013	9257	33.71	rural	12381	45.08
2017	9470	34.48	urban	15083	54.92
2021	8737	31.81	CPC (%)	27464	
age	27464	46.33(13.91)	non-CPC	24716	89.99
sex (%)	27464		CPC	2748	10.01
F	15037	54.75	work (%)	27464	
M	12427	45.25	unworking	9311	33.90
Edu (%)	27464		working	18153	66.10
primary and below	9246	33.67	sector (%)	18153	
high school	13791	50.21	private sector	15294	84.25
bachelor and above	4427	16.12	state sector	2859	15.75
Marital (%)	27464		area (%)	27464	
unmarried	5013	18.25	northeastern	2348	8.55
married	22451	81.75	eastern	10134	36.90
S-SES	27464	2.23(0.92)	central	7398	26.94
Income	27464	67947.53 (73135.16)	western	7584	27.61

*Notes*: (1) To achieve cross-year comparisons, standardization processing was applied to the indicators of sense of security and digital inclusion.

(2) The ranges for sense of security, life experiences, and digital inclusion were 1–4, 0–1, and 0–6, respectively.

(3) Standard deviations in parentheses for continuous variables.

### 3.3 Statistical methods

#### 3.3.1 Baseline analysis.

Given that sense of security is a continuous variable, we use OLS to test the relationships among life experiences, digital inclusion, and sense of security. The specific equation is:


$$Y_{i}=\alpha_{0}+\alpha_{1}X_{i}+{\alpha_{2}Controls}_{i}+\mu_{i}$$ (1)

where $Y_{i}$ is sense of security, X is life experiences or digital inclusion, and *Controls* are all covariates.

Additionally, another core issue is whether the impact of digital inclusion or life experiences on sense of security has increased, decreased, or remained unchanged over time. So, we construct the interaction terms between the independent variables and year dummy variables to explore the evolution of the effects. The equation is:


$$Y_{i}=\alpha_{0}+\alpha_{1}X_{i}+\alpha_{2}year2017+\alpha_{3}year2021+\alpha_{4}X_{i}\times year2017+\alpha_{5}X_{i}\times year2021+\alpha_{6}{Controls}_{i}+\delta_{i}$$ (2)

where the year 2013 serves as the reference year, with dummy variables representing the data from 2017 and 2021 (year2017, year2021). The two dummy variables allow the model to have different intercepts in different years, effectively addressing the issue of different distributions of the overall population in different periods [32]. Additionally, the model includes the interaction terms between the independent variables and the year dummy variables ($X_{i}\times year2017,\;\;X_{i}\times year2021$). Their estimated coefficients reflect the changes in the impact of digital inclusion or life experience in 2017 and 2021 compared with 2013. Most existing studies are based on the estimated coefficients of the independent variables ($\alpha_{1}$) to judge the impact of $X_{i}$, which does not assess the pattern of change. However, the estimated coefficients of the interaction terms ($\alpha_{4},\alpha_{5}$) may truly reflect the changing trend of the impact of $X_{i}$. Therefore, this study focuses on $\alpha_{4},\alpha_{5}$, which is also the focus of this study.

#### 3.3.2 Dominance analysis.

Dominance Analysis proposed by Budescu (1993), is a statistical method used to estimate the relative importance of multiple correlated independent variables in their impact on the dependent variable [34]. The basic idea is to use stepwise regression to determine the best regression model, and by comparing all possible sub-models, decompose the variance of the dependent variable according to the relative importance of each independent variable, represented as a percentage of the contribution rate. The equation is:


CXi(k)=∑(ρYXiXh2−ρYXh2)/(P−1k) (3)

where $X_{h}$ represents any subset of another *k* variables excluding $X_{i}$, $C_{X_{i}}^{\left(k\right)}$ represents the average contribution after the independent variable $X_{i}$ is added to a sub-model containing *k* independent variables, $C_{X_{i}}$ is obtained by averaging all the *P* sub-models’ $C_{X_{i}}^{\left(k\right)}$.

The average contribution of the variable X_*i*_ to the explanation of dependent variable is:


$$C_{X_{i}}=\sum_{k=0}^{P-1}C_{X_{i}}^{\left(k\right)}/P\mathrm{\backslash ]}$$ (4)

where *P* represents the number of independent variables; $C_{X_{i}}$ indicates the *k* independent variables (*k* = 0, 1, 2, …, *P*-1) included in the sub-model after excluding $X_{i}$. The relative importance of each independent variable is estimated by comparing the sizes of $C_{X_{i}}^{\left(k\right)}$.

## 4. Results

### 4.1 Influence of life experiences on sense of security

#### 4.1.1 The baseline model results.

To examine the influence of life experiences and its evolution, we conduct OLS on three datasets, including single-wave, two-wave, and three-wave data. This analytical strategy can not only compare the results from different years but also test the model robustness by changing different samples. As shown in Table 3 Models 1−5, after controlling for covariates, life experiences were significantly associated with sense of security in five datasets, indicating that the impact of life experiences is very significant and stable, which is consistent with the empirical results of other scholars’ research [2]. Compared with Models 1−3, which use single-wave data, the regression coefficient in Model 4 with a mixed cross-sectional data is relatively smaller, and the fit index(R^2^) is greater, indicating that the use of mixed cross-sectional data can increase the accuracy and effectiveness of the results. H 1−1 is verified.

**Table 3:** Results of life experiences affecting sense of security.

variables	Model1	Model 2	Model 3	Model 4	Model 5
	2013	2017	2021	2017&2021	2013-2021
*LE*	−0.467^***^	−0.316^***^	−0.406^***^	−0.295^***^	−0.407^***^
	(0.03)	(0.02)	(0.03)	(0.02)	(0.03)
year2017×life experiences					0.081^*^
					(0.03)
year2021×life experiences				−0.144^***^	−0.070^+^
				(0.03)	(0.04)
year2017					0.034^**^
					(0.01)
year2021				0.271^***^	0.308^***^
				(0.01)	(0.01)
covariates	Y	Y	Y	Y	Y
AIC	9966	13212	10926	24209	34545
BIC	10073	13326	11039	24350	34710
R^2^	0.173	0.078	0.068	0.128	0.155
N	9257	9470	8737	18207	27464

*Notes*: + *p* < 0.1,

*p < 0.05,

**p < 0.01,

***p < 0.001.

Existing research mostly assesses the effect size of direct experiences on sense of security based on single regression coefficients. However, due to the lack of longitudinal comparative analysis, it is not possible to accurately determine the evolving characteristics of the impact of life experiences. As shown in Models 1−3 in Table 3, the regression coefficients for life experiences were −0.462 in 2013, −0.316 in 2017, and −0.406 in 2021. Although the absolute value of the coefficients shows an overall downward trend, it cannot be concluded that the impact of life experiences has decreased. Further tests are needed to determine whether the reduction is statistically significant, that is, to examine whether the interaction term in Model 5 is significant. The regression coefficient for the interaction term (life experiences×year2017) is significantly positive (*β* = 0.081, *p* < 0.05), opposite to the direction of the main effect (*β* = −0.407, *p* < 0.001), indicating that compared with 2013, the negative impact of life experiences gradually weakened in 2017. The regression coefficient for the interaction term (life experiences×year2021) is marginally significantly negative (*β* = −0.070, *p* < 0.1), which is consistent with the direction of the main effect (*β* = −0.407, *p* < 0.001), suggesting that compared with 2013, the negative impact of life experiences gradually strengthened in 2021. Although the degree of increase in 2021 (7%) is less than that in 2017 (8.1%), it does not indicate that the impact of life experiences has weakened.

Additionally, in Model 4, the regression coefficient for the interaction term (life experience×year2021) is −0.144 significantly at the 0.1% level, which is consistent with the direction of the main effect (*β* = −0.295, *p* < 0.001), indicating that compared with 2017, the negative impact of life experiences has increased in 2021. Thus, it can be concluded that from 2013 to 2021, the negative impact of negative life experiences first weakened but then strengthened, rejecting H2-2.

#### 4.1.2 Robustness test.

To ensure the robustness, we adopt the variable substitution method, which uses alternative indicators of sense of security and life experiences (*MLE*). These results are presented in the Supporting Information (Tables S1-S2 in S2 Data). The results are consistent with the baseline results. This finding shows that the conclusions are robust and reliable, thereby confirming the negative impact of the negative life experiences, however, this negative impact manifests differently over time.

### 4.2 Influence of digital inclusion on sense of security

#### 4.2.1 The baseline model results.

Similarly, as shown in Table 4 Models 1–5, after controlling for covariates, digital inclusion was significantly associated with sense of security in five datasets, indicating that the impact of digital inclusion is very significant and stable, which is consistent with the empirical results of other researches [23,26]. The regression coefficients in Models 4 and 5 using mixed data are larger than those in Models 1–3 using single-wave data, and the fit indexes (R^2^) also increase, indicating that the use of mixed cross-sectional data can make the model estimation results more accurate and effective.

**Table 4:** The results of digital inclusion affecting sense of security.

variables	Model1	Model 2	Model 3	Model 4	Model 5
	2013	2017	2021	2017&2021	2013-2021
DI	−0.028^***^	−0.024^***^	−0.015^***^	−0.029^***^	−0.046^***^
	(0.00)	(0.00)	(0.00)	(0.00)	(0.00)
year2017×digital inclusion					0.020^***^
					(0.00)
year2021×digital inclusion				0.018^***^	0.037^***^
				(0.00)	(0.00)
year2017					0.048^***^
					(0.01)
year2021				0.240^***^	0.286^***^
				(0.01)	(0.01)
covariates	Y	Y	Y	Y	Y
AIC	10224	13341	11105	24501	35015
BIC	10331	13455	11218	24642	35179
R^2^	0.150	0.066	0.049	0.113	0.140
N	9257	9470	8737	18207	27464

*Notes*: + *p* < 0.1,

*p < 0.05,

**p < 0.01,

***p < 0.001.

As shown in models 1−3 in Table 4, the regression coefficients for digital inclusion were −0.028 in 2013, −0.024 in 2017, and −0.015 in 2021, thus H2-1 is verified. Although these three estimated coefficients decrease sequentially, it cannot be concluded that the impact of digital inclusion has declined. Further tests are needed to determine whether the reduction in the three-wave data is statistically significant, that is, to examine whether the interaction term in Model 5 is significant. As shown in Model 5 in Table 4, the regression coefficient for the interaction term (digital inclusion×year2017) is significantly positive (*β* = 0.020, *p* < 0.001), opposite to the direction of the main effect of digital inclusion (*β* = −0.046, *p* < 0.001), indicating that compared with 2013, the negative impact of digital inclusion gradually weakened in 2017. The regression coefficient for the interaction term (digital inclusion×year2021) is marginally significantly negative (*β* = 0.037, *p* < 0.1), and opposite to the direction of the main effect (*β* = −0.046, *p* < 0.001), suggesting that compared with 2013, the negative impact of digital inclusion gradually weakened in 2021. Moreover, the degree of the weakening impact of digital inclusion in 2021 (3.7%) was greater than that in 2017 (2%).

Additionally, in Model 4, the regression coefficient for the interaction term (digital inclusion×year2021) is 0.018 significantly at the 0.1% level, opposite to the direction of the main effect (β = −0.029, p < 0.001), indicating that compared with 2017, the negative impact of digital inclusion has decreased in 2021. Thus, it can be concluded that from 2013 to 2021, the negative impact of digital inclusion gradually weakened, verifying H2-2.

#### 4.2.2 Robustness test.

In addition to some measurement biases, the endogeneity may interfere with robustness. First, there may be some unobservable or omitted variables; second, there may also be a “sample self-selection” issue, meaning that it is not the level of digital inclusion, but rather that individuals with different levels of digital inclusion inherently have significant differences. To ensure the reliability, the following robustness analyses were conducted: substitution of the dependent variable, and an instrumental variable (IV) model. These results are presented in the Supporting Information (Tables S3-S4 in S2 Data). The results are consistent with the baseline results, which shows that the conclusions are robust and reliable. Thus, it confirms that digital inclusion negatively impacts on sense of security, and this negative impact shows a decreasing trend over time.

#### 4.2.3 Moderating role of educational level.

As mentioned above, the negative impact of digital inclusion on sense of security shows a decreasing trend, which may stem from improvements in public digital literacy. Some scholars have reported that education level was the most significant antecedent factor affecting digital literacy in China [35]. To further explore the reasons for the decreasing negative impact of digital inclusion, this study also analyzes the differences of education.

**Fig 1:**
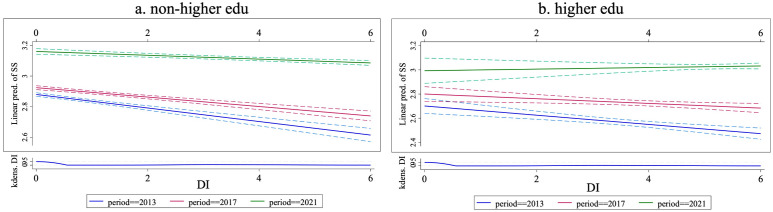
The educational differences in marginal effect of digital inclusion.

Fig 1 presents the differences between the non-higher educated and the higher educated groups in the relationship between digital inclusion and sense of security from 2013 to 2021. As shown in Fig 1a, for the non-higher educated group, the negative impact of digital inclusion continues to decrease (the negative slope becomes less steep). Fig 1b shows that for the higher educated group, the negative impact of digital inclusion decreases from 2013 to 2017 (the negative slope becomes less steep), and in 2021 it changes from negative to positive (the slope changes from negative to positive). These results, on the one hand, indicate that regardless of whether the group is the non-higher or higher educated group, digital inclusion has a significant and diminishing decreasing effect on sense of security, again confirming H2-1 and H2-2. On the other hand, they also suggest that improvements in education level or digital literacy can help mitigate the negative impact of digital inclusion on sense of security.

### 4.3 Life experiences VS digital inclusion: whose impact is stronger?

To determine the relative importance of life experiences and digital inclusion, we adopt the Dominance Analysis (DA) method. Table 5 Models 1, 4, and 7 present the relative importance (RI) results of life experiences and covariates based on CSS 2013, 2017, and 2021 datasets. Models 2, 5, and 8 present the RI results of digital inclusion and the covariates. Models 3, 6, and 9 present the RI results of life experiences, digital inclusion and covariates. On the one hand, the results from the same dataset (such as Models 1, 4, and 7) can clarify the relative magnitude of the impact of life experiences and digital inclusion in the same period. On the other hand, by comparing the relative importance rankings of different waves, we can also observe how the relative importance of life experiences or digital inclusion has changed over time. According to Ye et al. (2015), this study obtains the standardized contribution rates by standardizing the RI values, that is, the ratio of each variable’s RI to the total RI [36].

**Table 5:** The relative importance by dominance analysis.

variables	2013			2017			2021		
	Model1	Model 2	Model 3	Model 4	Model 5	Model 6	Model 7	Model 8	Model 9
	*LE*	*DI*	*Full*	*LE*	*DI*	*Full*	*LE*	*DI*	*Full*
*DI*		14.07% ***[4]***	12.18% ***[5]***		18.47% ***[1]***	15.42% ***[2]***		19.67% ***[2]***	13.69% ***[3]***
*LE*	15.82% ***[2]***		15.64% ***[2]***	19.20% ***[1]***		18.71% ***[1]***	32.10% ***[1]***		30.36% ***[1]***
age	5.24% [7]	5.44% [8]	4.08% [9]	12.81% [5]	12.60% [5]	9.68% [6]	6.89% [6]	7.16% [7]	5.01% [8]
Age^2^	5.08% [8]	5.74% [7]	4.09% [8]	13.98% [3]	14.71% [3]	10.88% [5]	7.73% [3]	8.32% [4]	5.50% [7]
sex	0.15% [15]	0.13% [15]	0.13% [16]	2.35% [9]	3.26% [8]	2.23% [8]	2.00% [11]	3.64% [9]	1.88% [10]
edu	15.44% [3]	14.27% [3]	12.35% [4]	17.30% [2]	14.72% [2]	13.03% [3]	21.85% [2]	21.17% [1]	17.62% [2]
marital	0.89% [14]	0.67% [14]	0.67% [15]	0.54% [14]	0.59% [14]	0.47% [15]	0.68% [12]	0.64% [13]	0.56% [13]
*hukou*	15.14% [4]	15.97% [2]	13.37% [3]	13.64% [4]	13.31% [4]	11.50% [4]	7.22% [5]	7.26% [5]	6.12% [5]
CPC	1.15% [11]	1.01% [11]	0.95% [12]	0.45% [15]	0.42% [15]	0.37% [16]	2.34% [8]	3.86% [8]	2.35% [9]
residence	20.40% [1]	21.21% [1]	18.30% [1]	10.48% [6]	10.93% [6]	9.22% [7]	6.50% [7]	7.17% [6]	5.58% [6]
work	2.15% [9]	2.12% [10]	1.89% [11]	0.92% [12]	1.03% [11]	0.84% [13]	0.43% [13]	0.44% [14]	0.39% [15]
sector	0.91% [13]	0.87% [12]	0.78% [14]	0.58% [13]	0.72% [12]	0.58% [14]	0.42% [14]	0.64% [12]	0.41% [14]
SES	2.11% [10]	4.35% [9]	2.18% [10]	1.73% [10]	4.27% [7]	1.96% [11]	7.47% [4]	15.92% [3]	6.98% [4]
income	7.22% [5]	6.65% [5]	6.24% [6]	2.52% [7]	2.17% [9]	2.13% [9]	2.11% [10]	1.99% [11]	1.70% [12]
Income^2^	7.22% [6]	6.65% [6]	6.24% [7]	2.52% [8]	2.17% [10]	2.13% [10]	2.11% [9]	1.99% [10]	1.70% [11]
Area	1.07% [12]	0.86% [13]	0.92% [13]	0.99% [11]	0.64% [13]	0.86% [12]	0.16% [15]	0.12% [15]	0.14% [16]
N	9257			9470			8737		

*Notes*: The percentage in each cell refers to the Relative Importance (RI) of each variable. First, the model R^2^ is decomposed into the shares of each regression term. The RI of the *j*th variable is its share in explaining the variance of the dependent variable. The numbers in brackets are the relative rankings of each variable.

As shown in Table 5 Models 1 and 2, the contribution rates of life experiences and digital inclusion to R^2^ were 15.82% and 14.07% respectively (both greater than 10%), ranking 2nd and 4th in 2013. This finding indicates that life experiences and digital inclusion have a relatively greater impact than other sociodemographic and socioeconomic factors do. As shown in Model 3, the explanatory power of digital inclusion (12.18%) is lower than that of life experiences (15.64%). Additionally, the RIs of the three variables—educational level, *hukou*, and residence—also exceeded 10%, indicating strong predictive power. Models 4 and 5 show that the explanatory powers of digital inclusion and life experiences in 2017 were 18.47% and 19.20% respectively (both greater than 10%), both ranking first. Compared with 2013, their contribution rates and relative importance rankings have increased, with digital inclusion showing a relatively larger increase. Model 6 shows that in 2017, the explanatory power of digital inclusion (15.42%) was still less than that of life experience (18.71%), whereas the ranking of digital inclusion rose from 5th in 2013–2nd in 2017. This indicates an upward trend in the impact of digital inclusion on sense of security. Furthermore, from Models 4–6, the RIs of education level and *hukou* also remain above 10%, indicating strong predictive powers. Models 7–8 show that the explanatory powers of digital inclusion and life experience in 2021 were 19.67% and 32.10% respectively, with a more significant increase than those in 2017. In Model 9, which includes all the variables, the influence of life experience (30.36%) still far exceeds that of digital inclusion (13.69%) and other factors. From an overall association perspective, the relative importance of factors has the following findings: First, from 2013 to 2021, compared with sociodemographic and economic indicators, digital inclusion and life experiences have always maintained a considerable advantages in explanatory power for sense of security. Second, although the explanatory powers of both digital inclusion and life experiences show the upward trend over time, life experiences is more important. Third, the contribution rates of education level and *hukou* have always remained at a high level, which is closely related to the upward mobility difficulties caused by the dual structure of Chinese society and the role of education in helping the lower strata move up. These results indicate that both digital inclusion and life experiences have strong predictive power, but life experience is more important, hence H3 is not verified.

In short, life experiences play a decisive role in the predicting sense of security. Although the explanatory power of digital inclusion is slightly weaker than that of life experiences, both are significantly stronger than are demographic and economic factors, and their influence has been steadily increasing over time. Due to the lag in the development of the Internet, digital inclusion itself has an innate longitudinal limitation. However, with the increasingly improved digital infrastructure in China, the level of the public’s digital inclusion in China has also shown an upward trend (2013–1.08; 2017–1.56; 2021–3.75; see Table 1), thus the role of digital inclusion in individuals’ daily lives will become more prominent. In addition, education level and *hukou* are important second only to life experience and digital inclusion, indicating that the government should consider the educational level and *hukou* in enhancing sense of security.

## 5. Discussion and conclusions

### 5.1 Research findings

This study utilizes mixed cross-sectional data from CSS 2013, 2017, and 2021, employing statistical methods such as the OLS, constructing interaction terms between core explanatory variables and year dummy variables, and Dominance Analysis (DA) to empirically investigate: a) the impact of life experiences and digital inclusion on sense of security and their evolutions over time, b) the relative importance of the two in explaining the changes in sense of security. The main findings are as follows: First, negative life experiences negatively affect sense of security, and this adverse impact shows a decreasing trend from 2013–2017, whereas an increasing trend from 2017–2021. Second, digital inclusion negatively affects sense of security, and this negative impact shows a decreasing trend over time, which is also influenced by an individual’s level of education. Third, from 2013–2021, life experiences have consistently played a decisive role in the formation of sense of security, demonstrating an advantageous position. In summary, development and changes in sense of security are influenced not only by individual digital behaviors but also constrained by direct experiences in real society. Specifically, over time, the negative impact of digital inclusion gradually weakens, whereas the negative impact of negative life experiences first decreases but then increases.

From the perspective of the experience shaping, this study explores the impact and its evolution of life experiences on sense of security, and finds that life experiences have a significant negative effect, which is consistent with existing research [37–39]. Moreover, this study expands the previous studies that were limited to victimization experiences and personal security, which is also the theoretical contribution and practical value of this study. The reasons for focusing on life experiences are as follow: first, although life experience does not shape an individual’s psychology as victimization experiences or major crisis events, it has a more distinct daily, continuous, and cumulative nature, and thus has stronger practical significance and guiding value. Second, the existing literature focusing on the impact of major emergencies neglect the potential threats of life difficulties in normal situations, which is also a typical problem in risk societies. In addition, one of the most valuable conclusions of this study is that it explores the evolution of the influence of life experience over time by constructing an interaction term between life experiences and survey-year dummy variables. That is, the negative impact of negative life experiences on sense of security shows a significant and stable decreasing trend from 2013−2017, and a significant increasing trend from 2017−2021, with the increase ratio in the second stage not exceeding the decrease ratio in the first stage. The slight rebound in the negative effect of negative life experiences from 2017–2021 may be due to the impact of real-life difficulties brought about by the COVID-19 pandemic. On this basis, the study speculates that the influence of negative life experiences on sense of security decreases over time in normal situations due to the large number of new and unknown risks created and spawned by global risk and technological development, leading to the failure of individuals’ existing prior experience and knowledge. The decreasing influence of real-life experiences on sense of security is inevitable.

According to the media construction theory, this study introduces the concept of “digital inclusion” and empirically analyzes its impact on sense of security. The results show that digital inclusion also has a significant negative impact on sense of security, and this negative influence tends to decrease over time. These findings not only enrich the existing perspective of information dissemination in the perception of online risk [14,24], but also strengthen the research foundation of digital inclusion in the field of social psychology. For scholars engaged in the risk perception and sense of security in the digital or online era, the media’s informational function (risk information production, information dissemination, etc.) is ultimately unavoidable, which is understandable. However, the fact focusing too much on the informational value of digital media would lead scholars to acknowledge the view that “digitalization equals informatization,” which clearly does not align with the social reality of the digital era. From a theoretical standpoint, the existing research conclusions regarding the perception of security in the digital era and the theoretical knowledge system are biased, and they certainly do not form academic theories that have both practical significance and explanatory value. In terms of the actual situation, with the deep integration of artificial intelligence, blockchain, and the Internet, the mutual embedding and structural integration of physical and virtual spaces, as well as real-life and digital lifestyles, have created a panoramic digital space. The impact of digital existence and lifestyle on individuals’ social psychology and behavior has long transcended the realm of information, thus making the entry point of digital inclusion more practically significant and academically promising.

Finally, this study also discusses the relative importance of life experiences and digital inclusion. Contrary to hypothesis, results show that from 2013–2021, life experiences have always remained dominant among the factors influencing sense of security. Even though the explanatory power of digital inclusion is increasing, the current level of digital inclusion cannot shake the decisive role of life experiences, which may be because the level of digital inclusion in China is low (see Table 2), with a mean of 2.09 and a range of 0–6. The study makes the following considerations: First, the decisive position of life experiences is in line with the theoretical core of Marxist practice view. Both social communication experience and media construction experience, they all need to be verified by individual practical activities [40]. In other words, digital elements should impact individuals’ psychology and behaviors through their real activities in the digital space, which is deeply integrated with real society. Second, if the governments want to achieve the goal of improving sense of security in the digital age, focusing on solving and improving people’s urgent and difficult life problems is an important measure. In addition, the differential analysis of education level shows that for the higher education group, the impact of digital inclusion on the sense of security changes from negative to positive over time, highlighting the importance of education. In contrast, existing research on social psychology in the digital age has not yet conducted in-depth theoretical discussions and empirical analyses on sense of security or risk perception from the perspective of digital inclusion. Therefore, although our findings are not sufficient to form conclusive facts, they can still provide resources for theoretical dialog and future work.

### 5.2 Limitations and future work

This study has several shortcomings. First, the mixed cross-sectional survey precludes drawing any causal conclusions and Longitudinal designs or experimental studies may provide more reliable and accurate explanations. Second, the measurements of sense of security need to be enriched; the current measurements, which are based on cognition, lack components related to emotional aspects. In the future, network text analysis that can capture emotional components could be added for a more comprehensive consideration. Third, the concept and operationalization of digital inclusion are still immature. With the full coverage of digital scenarios, the scope of digital inclusion has become broader and more comprehensive. However, due to the limitations of the CSS survey, the digital inclusion measured in this study is still insufficient, and it can be further enriched in the future. Finally, the measurement of life experiences is simply based on a cumulative count of negative events, which may oversimplify the nuanced nature of these experiences by not distinguishing between events of differing severity, duration, or frequency. Therefore, future research should enrich the dimensions of life experiences measurement or supplement qualitative methods to further clarify the complexity and differences of the impact of life experiences on the sense of security, thereby enhancing the scientific and rigorous nature of the research conclusions.

## Supporting information

S1 Data CSS data for this study. (CSV)

S2 Data The results of robustness test. (DOCX)
