# Supplementary material for: Life experiences vs digital inclusion: Factors influencing sense of security and their evolutions in China
Source: PLoS One. 2025 Aug 28;20(8):e0330667. doi: 10.1371/journal.pone.0330667 (PMC12393730; doi:10.1371/journal.pone.0330667)
Supplement: S2 Data — (DOCX) [file pone.0330667.s002.docx]

**The Results of Robustness test**

*4.1.2 Robustness test*

**Table S1**

The robustness test results of life experience affecting sense of security (dependent variable submission)

| Variables | Model1 | Model 2 | Model 3 | Model 4 | Model 5 |
| --- | --- | --- | --- | --- | --- |
|  | 2013 | 2017 | 2021 | 2017&2021 | 2013-2021 |
| Life experience | -0.446^***^ | -0.230^***^ | -0.310^***^ | -0.222^***^ | -0.391^***^ |
|  | (0.03) | (0.03) | (0.03) | (0.03) | (0.03) |
| $life experience\times year2017$ |  |  |  |  | 0.048^***^ |
|  |  |  |  |  | (0.03) |
| $life experience\times year2021$ |  |  |  | -0.098^*^ | 0.040 |
|  |  |  |  | (0.04) | (0.04) |
| year2017 |  |  |  |  | 0.142^***^ |
|  |  |  |  |  | (0.04) |
| year2021 |  |  |  | 0.249^***^ | 0.300^***^ |
|  |  |  |  | (0.01) | (0.01) |
| covariates | Y | Y | Y | Y | Y |
| AIC | 13090 | 16049 | 12995 | 29144 | 42384 |
| BIC | 13196 | 16163 | 13108 | 29284 | 42549 |
| R^2^ | 0.084 | 0.039 | 0.045 | 0.082 | 0.103 |
| N | 9063 | 9376 | 8649 | 18025 | 27088 |

Notes:（1）+ *p*<0.1，* *p*<0.05，***p*<0.01，****p*<0.001；（2）Covariates includes age, age2, sex, marital status, educational level, residence, hukou, CPC, work, income, ses, sector, area.

**Table S2**

The robustness test results of major life events (*MLE*) affecting sense of security (independent variable submission)

| variables | Model1 | Model 2 | Model 3 | Model 4 | Model 5 |
| --- | --- | --- | --- | --- | --- |
|  | 2013 | 2017 | 2021 | 2017&2021 | 2013-2021 |
| MLE | -0.284^***^ | -0.367^***^ | -0.230^***^ | -0.376^***^ | -0.300^***^ |
|  | (0.03) | (0.03) | (0.03) | (0.03) | (0.03) |
| $MLE\times year2017$ |  |  |  |  | -0.077^+^ |
|  |  |  |  |  | (0.04) |
| $MLE\times year2021$ |  |  |  | 0.147^***^ | 0.068 |
|  |  |  |  | (0.04) | (0.04) |
| year2017 |  |  |  |  | 0.071^***^ |
|  |  |  |  |  | (0.01) |
| year2021 |  |  |  | 0.221^***^ | 0.294^***^ |
|  |  |  |  | (0.01) | (0.01) |
| covariates | Y | Y | Y | Y | Y |
| AIC | 10182 | 13248 | 11088 | 24406 | 34956 |
| BIC | 10289 | 13363 | 11201 | 24546 | 35120 |
| R^2^ | 0.153 | 0.074 | 0.051 | 0.118 | 0.142 |
| N | 9255 | 9467 | 8737 | 18204 | 27459 |

Notes:（1）+ *p*<0.1，* *p*<0.05，***p*<0.01，****p*<0.001；（2）Covariates includes age, age2, sex, marital status, educational level, residence, hukou, CPC, work, income, ses, sector, area

*4.2.2 Robustness test*

(1) Re-estimation based on alternative indicators of the dependent variable.

**Table S3**

The robustness test results of digital inclusion (*DI*) affecting sense of security (dependent variable submission)

| Variables | Model1 | Model 2 | Model 3 | Model 4 | Model 5 |
| --- | --- | --- | --- | --- | --- |
|  | 2013 | 2017 | 2021 | 2017&2021 | 2013-2021 |
| *DI* | -0.031^***^ | -0.016^***^ | -0.007^**^ | -0.018^***^ | -0.038^***^ |
|  | (0.00) | (0.00) | (0.00) | (0.00) | (0.00) |
| $DI\times year2017$ |  |  |  |  | 0.021^***^ |
|  |  |  |  |  | (0.00) |
| $DI\times year2021$ |  |  |  | 0.011^**^ | 0.033^***^ |
|  |  |  |  | (0.00) | (0.00) |
| year2017 |  |  |  |  | 0.072^***^ |
|  |  |  |  |  | (0.01) |
| year2021 |  |  |  | 0.226^***^ | 0.298^***^ |
|  |  |  |  | (0.01) | (0.01) |
| covariates | Y | Y | Y | Y | Y |
| AIC | 13242 | 16099 | 13086 | 29277 | 42618 |
| BIC | 13349 | 16206 | 13192 | 29410 | 42774 |
| R^2^ | 0.068 | 0.034 | 0.035 | 0.076 | 0.095 |
| N | 9063 | 9376 | 8649 | 18025 | 27088 |

Notes:（1）+ *p*<0.1，* *p*<0.05，***p*<0.01，****p*<0.001；（2）Covariates includes age, age2, sex, marital status, educational level, residence, hukou, CPC, work, income, ses, sector, area.

(2) Endogeneity test: Re-estimation based on instrumental variables.

According to previous papers (Chen, 2013; Xi et al.,2020), we adopt two Instrumental variables of Digital Inclusion: Household communication expenditure and the Internet penetration rates of each province in 2010, which have been confirmed in their rationality.

In order to further verify the stability of the impact of digital inclusion on sense of security, the two-stage least squares (2SLS) method was utilized to incorporate the instrumental variable into the model for analysis. Table S4 presents the results of IV model.

**Table S4**

The results of digital inclusion affecting sense of security: Household communication expenditure and the Internet penetration rates as Instrumental variables

| Variables | Model1 | Model 2 | Model 3 | Model 4 | Model 5 |
| --- | --- | --- | --- | --- | --- |
|  | 2013 | 2017 | 2021 | 2017&2021 | 2013-2021 |
| *DI* | -0.307^***^ | -0.246^***^ | -0.122^***^ | -0.210^***^ | -0.267^***^ |
|  | (0.05) | (0.04) | (0.03) | (0.02) | (0.02) |
| $DI\times year2017$ |  |  |  |  | 0.053^***^ |
|  |  |  |  |  | (0.01) |
| $DI\times year2021$ |  |  |  | 0.070^***^ | 0.127^***^ |
|  |  |  |  | (0.01) | (0.01) |
| year2017 |  |  |  |  | 0.117^***^ |
|  |  |  |  |  | (0.02) |
| year2021 |  |  |  | 0.411^***^ | 0.520^***^ |
|  |  |  |  | (0.06) | (0.05) |
| covariates | Y | Y | Y | Y | Y |
| N | 9054 | 9303 | 8322 | 17625 | 26679 |
| First stage F | 48.68 | 60.42 | 25.99 | 67.44 | 95.34 |
| DWH *p* | 0.000 | 0.000 | 0.000 | 0.000 | 0.000 |

Notes:（1）+ *p*<0.1，* *p*<0.05，***p*<0.01，****p*<0.001；（2）Covariates includes age, age2, sex, marital status, educational level, residence, hukou, CPC, work, income, ses, sector, area
